# Dual-Functional Carbon Residue Derived from Co-Pyrolysis of Iron Sludge and Biochar for Synergistic Adsorption and Catalytic Oxidation

**DOI:** 10.3390/molecules31132374

**Published:** 2026-07-06

**Authors:** Zhipeng Li, Gangzheng Sun, Hao Zhang, Yiwei Xiang, Weikun Zhang, Guoying Pang, Siyu Wei, Nanxiang Deng, Tan Meng

**Affiliations:** 1 School of Mechanical and Power Engineering, Tianjin Renai College, Tianjin 301636, China; 2 Shengli Oilfield Company, Sinopec, Dongying 257000, China; 3 School of Environmental Science and Engineering, Tianjin University, Tianjin 300072, China

**Keywords:** adsorption, carbon residue, catalytic, iron, persulfate, sulfamethoxazole

## Abstract

The persistence of refractory organic pollutants (e.g., antibiotics) in aquatic environments necessitates efficient and sustainable remediation strategies. In this study, a circular economy approach was adopted to convert iron sludge into a value-added carbon residue (CR) composite via one-step co-pyrolysis. The resulting material was designed as dual-functional, enabling synergistic pollutant removal through adsorption and catalytic oxidation. Experimental results demonstrated that the CR composite effectively adsorbed and degraded organic pollutants. The primary adsorption sites were attributed to surface functional groups, porous structure, and electrostatic interactions. Meanwhile, iron species, surface functional groups, and persistent free radicals facilitated the generation of singlet oxygen (^1^O_2_) and hydroxyl radicals (·OH), which in turn promoted pollutant degradation. The CR/PDS system exhibited excellent performance in real wastewater remediation, which was attributed to the high interference resistance of ^1^O_2_. Furthermore, the application of CR did not pose any significant environmental risk in aqueous solutions. Taken together, these findings present a novel material for pollutant removal and provide a cost-effective strategy for the valorization of waste iron sludge.

## 1. Introduction

The efficient removal of organic pollutants from aquatic environments remains a major challenge in environmental science and engineering [1,2]. Emerging contaminants, particularly antibiotics, have attracted considerable attention due to their environmental persistence, bioaccumulation potential, and associated risks to ecological and human health [3]. Conventional methods, including physical adsorption and biological treatment, are often limited by incomplete removal, secondary pollution, or insufficient mineralization of refractory compounds. In contrast, sulfate radical-based advanced oxidation processes (SR-AOPs) have shown great promise, attributed to the high redox potential, wide pH adaptability, and relatively long half-life of sulfate radicals (·SO_4_^−^) [4,5]. The activation of persulfates—peroxydisulfate (PDS) or peroxymonosulfate (PMS)—is central to SR-AOPs, necessitating efficient, stable, and low-cost heterogeneous catalysts for practical application [6].

Carbon-based materials, such as biochar (a porous carbon derived from oxygen-limited pyrolysis of biomass), have been widely studied as adsorbents and catalyst supports [7,8]. Biochar possesses not only a high surface area and tunable pore structure but also abundant surface oxygen functional groups (e.g., -COOH, -OH), which contribute to its adsorption capacity and provide sites for further functionalization [9,10]. However, pristine biochar typically exhibits limited catalytic activity. Modification strategies, including the incorporation of transition metals, are commonly employed to enhance catalytic performance. Iron-based materials (e.g., zero-valent iron and iron oxides) have been extensively investigated as effective persulfate activators [11,12,13]. Nevertheless, nano-iron particles tend to agglomerate and oxidize, and may cause secondary pollution through iron leaching, thereby hindering large-scale application. Supporting iron species on a porous carbon matrix can mitigate these issues: the carbon carrier helps disperse and stabilize the iron species, while its sp^2^-hybridized carbon framework, defects, or persistent free radicals can synergistically activate persulfates, often resulting in a synergistic catalytic effect [1].

Selecting an appropriate and low-cost iron source is critical for scaling up such composite materials. Iron-rich sludge from water treatment plants—a byproduct of coagulation processes using iron-based coagulants such as polyferric sulfate or ferric chloride—represents a promising low-cost iron precursor [14]. This sludge consists primarily of amorphous iron oxyhydroxides along with residual organic and inorganic impurities [15]. Conventional disposal methods (e.g., landfilling) incur significant costs and pose environmental risks. Transforming this end-of-pipe waste into a resource aligns with circular economy principles and sustainable development goals. The iron in this sludge, present in a reactive amorphous form, can be repurposed as an iron precursor for catalytic materials, enabling a “waste-treat-waste” strategy that reduces both material costs and sludge disposal burdens [16,17,18].

Accordingly, this study proposes an innovative waste-to-resource approach that converts iron sludge and waste biomass into an iron-modified carbon-based composite via one-step co-pyrolysis. The resulting material is designed to achieve synergistic adsorption and catalytic oxidation of organic pollutants. The specific objectives are (1) to characterize the physicochemical properties of the composite; (2) to evaluate its adsorption and catalytic performance in pollutant removal; (3) to investigate the underlying mechanisms of pollutant removal and oxidant activation; and (4) to assess its potential for wastewater remediation. This work aims to provide a feasible pathway for the value-added reuse of iron sludge while developing a low-cost, dual-functional material for the treatment of refractory organic wastewater.

## 2. Results and Discussion

### 2.1. Adsorption Ability of CRs

**Figure 1:**
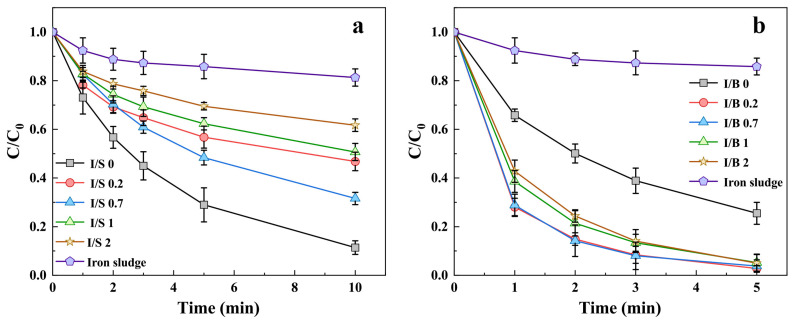
The adsorption of methylene blue by CRs made from iron sludge and sawdust (**a**), [CR] = 1 g/L, [Methylene blue] = 25 μM, and pH = 7; the adsorption of methylene blue by CRs made from iron sludge and biochar (**b**), [CR] = 1 g/L, [Methylene blue] = 25 μM, and pH = 7.

The adsorption kinetics of methylene blue (MB) by CRs produced with different weight ratios of iron sludge to biomass or biochar were investigated. Methylene blue removal by CR pyrolyzed from pure iron sludge was limited (less than 15%). In contrast, the removal efficiency increased with the addition of sawdust, reaching 39%, 49%, 68%, 55%, and 88% for I/S 2, I/S 1, I/S 0.5, I/S 0.2, and I/S 0, respectively, within 10 min (Figure 1a). This indicated that a higher sawdust content generally improved pollutant adsorption, although I/S 0.7 exhibited the best performance. A similar trend was observed for the adsorption of methylene blue by CRs derived from iron sludge and biochar. The increase in biochar addition also enhanced adsorption performance. Notably, I/B 0.2 and I/B 0.7 showed excellent methylene blue removal, even outperforming I/B 0 (Figure 1b). This improvement was attributed to the introduction of iron sludge, which altered the physicochemical properties of biochar and resulted in a better adsorbent.

To compare the reaction rates of different CRs for methylene blue adsorption, pseudo-first-order reaction kinetics were applied. The corresponding rate constants are listed in Table S1. Among the CRs, I/B 0.7 exhibited the highest observed rate constant (k_obs_ = 0.1696 min^−1^), which was higher than that of the other CRs. Compared with CRs derived from the pyrolysis of sawdust, the I/B series demonstrated exceptional pollutant adsorption capability. Based on the adsorption performance for methylene blue, I/S 0, I/S 0.7, I/B 0.7, and pure iron sludge were selected for subsequent experiments.

In addition to methylene blue, the CR derived from iron sludge and biochar also exhibited superior adsorption performance for SMX. Specifically, I/B 0.7 achieved a 90% removal rate of SMX within 30 min (Figure S1), which was higher than that of I/S 0 (65%) and I/S 0.7 (40%). In contrast, the CR produced solely from iron sludge showed no significant effect on SMX adsorption. These results indicate that iron sludge alone is not an effective raw material for adsorbent preparation.

### 2.2. Catalytic Performance of CRs

**Figure 2:**
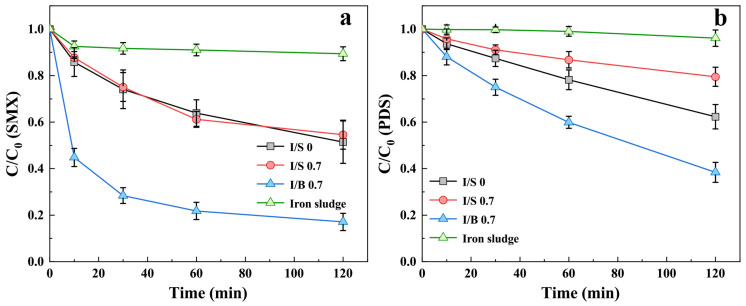
The SMX degradation (**a**) and PDS decomposition (**b**) in the CR/PDS system. [CR] = 1 g/L, [PDS] = 1 mM, [SMX] = 10 μM, and pH = 7.

To evaluate the catalytic activity of the prepared CRs, peroxydisulfate (PDS) was employed as the oxidant in this system. As shown in Figure 2a and Table S2, the I/B 0.7 composite exhibited outstanding performance in the degradation of sulfamethoxazole (SMX), achieving nearly 80% removal within 120 min. In contrast, I/S 0 and I/S 0.7 achieved only 40–42% degradation under identical conditions. The pyrolyzed product derived from iron sludge alone, despite the presence of iron and other organic constituents, displayed negligible catalytic activity. This observation is likely attributable to the insufficient organic content in the raw sludge, which hinders the formation of a porous, biochar-like matrix during pyrolysis.

To further assess the catalytic capacity of the CRs, the decomposition kinetics of PDS were monitored, and the results are presented in Figure 2b. I/B 0.7 facilitated PDS decomposition to extents of 40% and 62% within 120 min and 180 min, respectively. By comparison, the PDS decomposition efficiencies over 180 min were 38%, 21%, and 4% for I/S 0, I/S 0.7, and pyrolyzed iron sludge, respectively. These trends are closely consistent with the observed SMX degradation efficiencies, collectively confirming the superior catalytic performance of I/B 0.7 relative to the other materials. This enhanced activity may stem from the incorporation of biochar, which promotes better dispersion and anchoring of iron species derived from the iron sludge, thereby increasing the density and accessibility of active sites. The underlying reaction mechanisms will be elaborated in the subsequent sections.

### 2.3. Reactive Oxygen Species Detection

**Figure 3:**
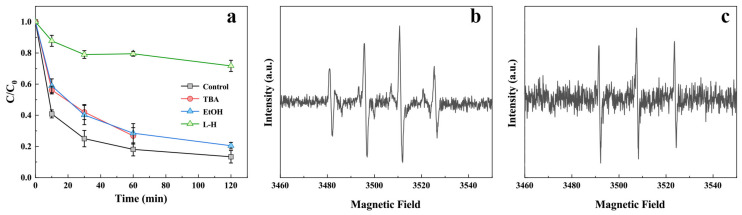
The effect of quenching agents in SMX degradation (**a**), [I/B 0.7] = 1 g/L, [PDS] = 1 mM, [SMX] = 10 μM, and pH = 7; EPR spectra of PDS activation by I/B 0.7 (**b**,**c**), [CR] = 0.5 g/L, [PDS] = 1 mM, and pH = 7.

To identify the reactive oxygen species (ROS) involved in SMX degradation, quenching experiments and electron paramagnetic resonance (EPR) spectroscopy were conducted. Previous studies have reported the generation of hydroxyl radicals (·OH) and sulfate radicals (SO_4_•^−^) in the CR/PDS system and their contributions to SMX degradation. As shown in Figure 3a, the addition of tert-butyl alcohol (TBA), a scavenger of ·OH, slightly reduced SMX degradation from 87% to 79%, suggesting that ·OH may be involved in the degradation process. Meanwhile, ethanol (EtOH), which effectively scavenges both ·OH and •SO_4_^−^, also decreased SMX degradation to an extent similar to that observed with TBA, indicating that the role of •SO_4_^−^ in SMX degradation was minor.

To further investigate the role of singlet oxygen (^1^O_2_), L-histidine (L-H) was employed in the CR/PDS system. Figure 3a shows that SMX degradation was significantly suppressed, decreasing from 87% to 29% within 120 min, indicating that ^1^O_2_ may play a dominant role in the CR/PDS system. EPR spectroscopy was performed using DMPO as a spin-trapping agent to detect ·OH generated in the CR/PDS system. As depicted in Figure 3b, a characteristic quartet peak with a 1:2:2:1 pattern—attributable to the DMPO-OH adduct—was observed in the CR/PDS system as the reaction proceeded. Furthermore, when TEMP was used as the trapping agent, the EPR spectrum exhibited distinct three-line signals of equal intensity, corresponding to the TEMP-^1^O_2_ adduct [19,20]. Based on these findings, it can be concluded that both ·OH and ^1^O_2_ contribute to SMX degradation, with ^1^O_2_ being the dominant reactive oxygen species.

### 2.4. Possible Mechanism of SMX Removal

#### 2.4.1. Adsorption Mechanism of CR

To elucidate the mechanism of SMX adsorption by CR, FTIR and XPS analyses were performed to identify changes in functional groups. As depicted in Figure S2, the signals at 1640 cm^−1^ and 3450 cm^−1^, corresponding to C=O and -OH groups, respectively, changed significantly after SMX adsorption. Meanwhile, the O 1s XPS spectrum indicated that the content of C=O decreased from 32.5% to 28.2%, and the proportion of -OH decreased from 21.2% to 15.8% following SMX adsorption. These changes can be attributed to the reaction between CR and SMX, which substantially consumed these functional groups.

As a porous material, pore filling also represented an important mechanism for pollutant removal [21,22]. The results in Table S3 show that both pore volume and pore size decreased after SMX adsorption. Furthermore, EDS results indicated that the contents of carbon (C) and sulfur (S) increased, which can be attributed to the adsorption of SMX on the surface (Table S4). Table S5 shows that the pH_pzc_ of the CRs was higher than 8.89, indicating that the material carries a positive charge in water at pH 7. The pKa values of SMX are 1.6 and 5.7, indicating that SMX predominantly exists as negatively charged ions (SMX^−^) at pH 7. Therefore, it can be inferred that electrostatic attraction occurs between the positively charged CR and the negatively charged SMX ions, thereby facilitating the adsorption and removal of the pollutant.

#### 2.4.2. Degradation Mechanism in CR/PDS System

**Figure 4:**
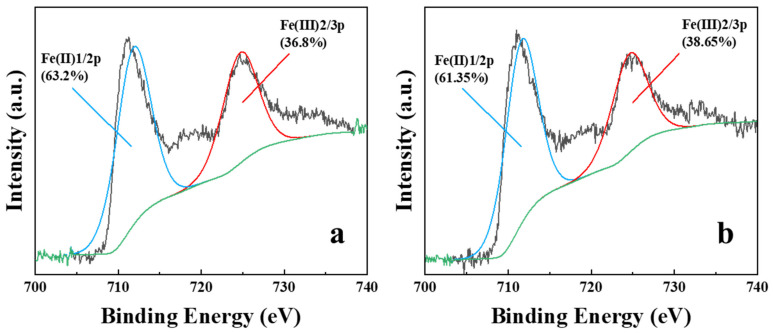
XPS (Fe 2p) spectra of I/B 0.7 before (**a**) and after (**b**) reaction with PDS.

Previous studies have established that iron doping is an essential modulator of material performance in oxidative activation. To elucidate the degradation mechanism of SMX in the CR/PDS system, XPS analysis was performed in this study. As shown in Figure 4, the content of Fe(II) (2p) decreased from 63.2% at 0 min to 61.35% at 30 min, while the content of Fe(III) (3/2p) increased significantly after reaction with PDS. This change in Fe(II) and Fe(III) contents indicates that iron plays a pivotal role in PDS activation and SMX degradation.

To determine the contribution of iron released from CR to PDS activation and SMX degradation, ICP-OES was used to measure the iron concentration in the solution matrix. The results showed that the iron concentration was less than 0.1 mg/L within 30 min, indicating that free iron ions are not the essential active sites in the CR/PDS system.

**Figure 5:**
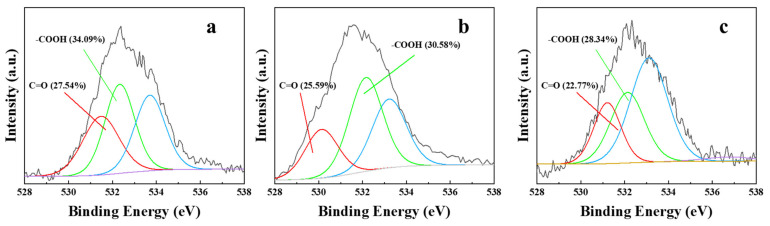
XPS (O 1s) spectra of I/B 0.7 before reaction (**a**), after SMX adsorption (**b**), and after reaction with PDS (**c**).

Furthermore, the O 1s XPS spectra of CR (Figure 5) showed that the content of C=O decreased from 27.54% to 22.77%, and the -OH content decreased from 34.09% to 28.34%. Compared with the results obtained after SMX adsorption alone, the introduction of PDS further reduced the proportion of these functional groups. This suggests that both C=O and -OH groups serve as reactive sites in adsorption and degradation reactions.

A chemical titration was conducted using phenylhydrazine (PH) and benzoic anhydride (BA) to selectively deactivate ketone (C=O) groups and -OH groups, respectively, without altering other structural features [23,24]. The CR samples after deactivation by PH and BA were subsequently used to activate PDS and degrade SMX, and the results are presented in Figure S3. It was observed that the degradation efficiency of SMX and the activation efficiency of PDS decreased significantly after treatment with PH and BA, with PH exhibiting a stronger inhibitory effect. The EPR results showed that treatment with PH and BA also led to weakened peak intensities for ^1^O_2_ and ·OH. Therefore, it can be concluded that the presence of C=O and -OH groups in CR is responsible for PDS activation and SMX degradation.

In addition to functional groups, persistent free radicals (PFRs) present in CR also serve as active sites for PDS activation. The role of PFRs in PDS activation and SMX degradation was investigated using KI as a scavenger. The EPR results showed that KI treatment decreased the PFR intensity from 6.052 × 10^5^ spins/g to 4.019 × 10^5^ spins/g (Table S6), indicating effective inhibition of PFRs. The results in Figure S4 show that KI treatment reduced PDS activation from 62% to 38% within 120 min, while SMX degradation dropped from 86% to 72%. This indicates that PFRs play a role in facilitating both SMX degradation and PDS activation.

Raman spectra (Figure S5) revealed peaks at 1342 cm^−1^ and 1595 cm^−1^, which were assigned to the D and G bands of all specimens, respectively. The disorder-induced D band corresponds to structural imperfections, whereas the in-plane vibrational G band is associated with sp^2^-hybridized graphitic carbon atoms. The intensity ratio (I_D_/I_G_) is commonly used to estimate the defect density in carbon materials. To explore the role of defects, the performance of CR was tested after treatment with EDTA-2Na, which reacts with surface defects to form formaldehyde [25,26]. The detection of formaldehyde in the CR suspension with EDTA-2Na confirmed the presence of surface defects on CR. After treatment with EDTA-2Na, the I_D_/I_G_ ratio decreased from 0.998 to 0.961. However, as shown in Figure S6, the EDTA-2Na-treated CR exhibited similar PDS activation and SMX degradation relative to untreated CR. Collectively, these results strongly indicate that surface defects make an insignificant contribution to the oxidative sites on CR.

### 2.5. Reusability of CR

**Figure 6:**
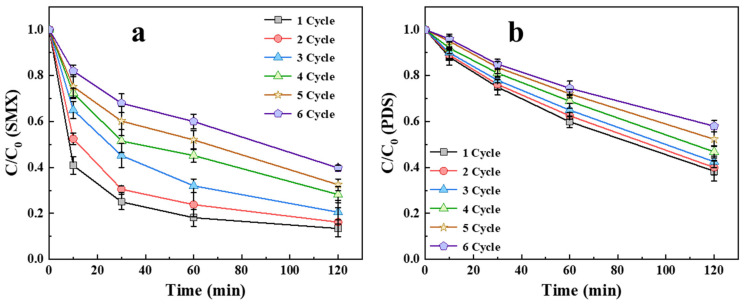
The reusability of CR in SMX degradation (**a**), [I/B 0.7] = 1 g/L, [PDS] = 10 mM, [SMX] = 10 μM, and pH = 7; The reusability of CR in PDS activation (**b**), [I/B 0.7] = 1 g/L, [PDS] = 10 mM, [SMX] = 10 μM, and pH = 7.

To evaluate the practical applicability of CR, reusability and stability experiments were conducted, as depicted in Figure 6. The results showed that the degradation performance remained nearly unchanged during the first two cycles, with a degradation rate exceeding 85% within 120 min. A downward trend was observed from the third cycle onward, and the degradation rate dropped to approximately 60% by the sixth cycle. Furthermore, as shown in Figure S8, regeneration of the CR used in the sixth cycle via washing with petroleum ether increased the SMX degradation efficiency from 60% to 75%. This suggests that saturation adsorption of residual oil onto the CR may mask its surface, thereby further influencing its catalytic performance.

### 2.6. Application in Wastewater Treatment

**Figure 7:**
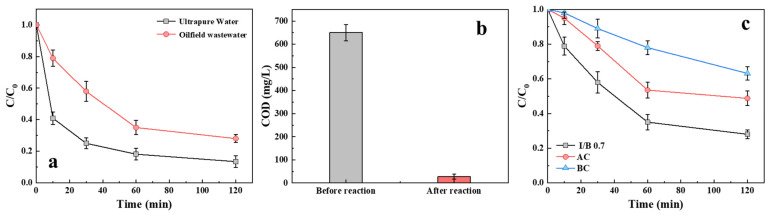
The SMX degradation of the I/B 0.7/PDS system in wastewater treatment (**a**), [CR] = 1 g/L, [PDS] = 1 mM, [SMX] = 10 μM, and pH = 7; The removal of COD by the I/B 0.7/PDS system (**b**), [CR] = 1 g/L, [PDS] = 1 mM, [SMX] = 10 μM, and pH = 7; The comparison of different materials in pollutant degradation (**c**), [Materials] = 1 g/L, [PDS] = 1 mM, [SMX] = 10 μM, and pH = 7.

To assess the potential of the CR/PDS system for environmental remediation, produced water from an oilfield was selected as a target processing sample. Compared with the ultrapure water matrix, the degradation of SMX in the wastewater decreased from 87% to 72% within 120 min (Figure 7). This observed decline can be attributed to several factors. First, the presence of interfering ions in wastewater, such as Cl^−^, HCO_3_^−^, and PO_4_^3−^, can affect SMX degradation by reacting with reactive oxygen species, leading to the formation of species with lower oxidation capacity. Furthermore, the high concentration of organic matter in wastewater can react with reactive oxygen species, resulting in unnecessary consumption of oxidants. Additionally, residual oil may partially occupy or block certain active sites on the catalyst surface through competitive adsorption, thereby reducing the accessibility of persulfate and target pollutants to those sites and consequently lowering the generation efficiency of reactive species.

### 2.7. Comparison with Other Materials

Activated carbon (AC) and biochar (BC) were comparatively evaluated against I/B 0.7 for SMX degradation. As illustrated in Figure 7, I/B 0.7 exhibited significantly superior degradation performance in SMX degradation, achieving a degradation efficiency of 72% within 120 min—substantially higher than the 51.2% and 36.8% attained by AC and BC. This can be attributed to the fact that I/B 0.7/PDS could produce more reactive oxygen species, and the main ROS was ^1^O_2_, which had higher anti-interfering ability against the interfering ions and dissolved organic matter [27,28]. The superior performance of the I/B 0.7/PDS system under identical reaction conditions demonstrates its distinct catalytic advantage over conventional activation materials.

### 2.8. Toxicity Test of Materials

Previous research has demonstrated that polycyclic aromatic hydrocarbons (PAHs) can be produced during the pyrolysis of biomass and sludge. PAHs are known toxic substances that may endanger soil microorganisms, raising concerns about potential environmental risks to soil and groundwater. Therefore, the Soxhlet extraction method was used to extract potential PAHs from the CR, and gas chromatography–mass spectrometry (GC-MS) was employed for their detection. The results showed that no PAH components were present in the CR.

In addition, metal ions contained in the sludge may leach from the resulting material and pose a threat to the aqueous matrix. The results presented in Figure S7 show that 1.268 mg/L of Fe was detected in a 1 g/L CR suspension. The low iron content may be attributed to the cleaning process after pyrolysis, which removed iron ions that were not firmly attached to the material. To assess the environmental impact of the co-pyrolysis material, a bacterial luminescence assay was conducted to evaluate the toxicity of the CRs. The results depicted in Figure S8 reveal that the leachate from CR had no significant effect on bacterial luminescence compared with control conditions. Notably, despite the iron leached from CR, bacterial luminescence was not suppressed [29,30]. This phenomenon is likely attributable to the low iron concentration, which was insufficient to affect bacterial bioluminescence. Furthermore, previous studies (including our own) have demonstrated the absence of other heavy metals and dioxins in the carbon residues produced during CR generation. Consequently, these findings suggest that CR does not yield toxic components. Therefore, the utilization of the resulting CR for wastewater remediation is unlikely to pose environmental threats to aquatic ecosystems.

## 3. Materials and Methods

### 3.1. Materials

Iron sludge was collected from urban waterworks in Tianjin, China. Sawdust (biomass) and biochar were purchased from a company (Uncle Zhao’s Farm, Zhengzhou) in Henan Province, China. Potassium peroxydisulfate (PDS) was obtained from HEOWNS Co., Ltd. (Tianjin, China). All other analytical-grade chemicals were supplied by Heowns Corporation (Tianjin, China) and Kermel Industrial Corporation (Tianjin, China). Nanopure water (resistivity > 18.2 MΩ·cm) was produced using a Merck Milli-Q Reference system (Merck KGaA, Darmstadt, Germany).

### 3.2. Pyrolysis Experiments

The pyrolysis experiments were carried out as follows. First, the iron sludge was crushed into small particles and then mixed with sawdust or biochar at various weight ratios (0:1; 0.2:1, 0.7:1; 1:1, 2:1, and iron sludge only). The resulting mixture was placed into a crucible, covered with a lid, and pyrolyzed in a muffle furnace without a nitrogen gas flow. The furnace was heated to the target temperature (700 °C) at a ramp rate of 10 °C/min, and the temperature was maintained for 2 h. After pyrolysis, the resulting carbon residue (CR) was washed five times with 0.1% nitric acid, followed by rinsing with ultrapure water. The washed CR was then ground to a uniform particle size, and stored in airtight bags until further use. CR produced from iron sludge and sawdust was designated as I/S n, and CR produced from iron sludge and biochar was designated as I/B n, where n represents the weight ratio of iron sludge to sawdust or biochar (i.e., n:1). Iron sludge means the carbon residue was made by the pyrolysis of iron sludge only.

### 3.3. Characterization

Surface functional groups were analyzed using Fourier transform infrared spectroscopy (FTIR; HORIBA EMAX, HORIBA, Kyoto, Japan). Specific surface area was determined by a surface area analyzer (ASAP2020M, Micromeritics, Norcross, GA, USA) at 77 K. Raman spectra of the CR samples were acquired using a Raman system (inVia plus, Renishaw, Gloucestershire, England) with an excitation wavelength of 532 nm. Electrical conductivity was measured with a four-point probe system. Surface chemical compositions were examined by X-ray photoelectron spectroscopy (XPS; Escalab 250Xi, Waltham, MA, USA). Morphological features were observed using scanning electron microscopy (SEM; FEI Apreo S, Waltham, MA, USA), and elemental compositions were analyzed by energy-dispersive spectroscopy (EDS) coupled with the same SEM system. Generated radical species were monitored by electron spin resonance (ESR) spectroscopy (EMXPLUS10/12, Bruker, Bremen, Germany) at room temperature under the following conditions: resonance frequency of 9.84 GHz, microwave power of 20.00 mW, modulation frequency of 100.00 kHz, and modulation amplitude of 2.00 G.

### 3.4. Experimental Procedures

All batch experiments were conducted in 100 mL screw-sealed bottles containing 50 mL of solution placed on a magnetic stirrer (JB-11, INASE Scientific Instrument CO., Shanghai, China) at room temperature. To evaluate the catalytic performance of the prepared CR in advanced oxidation processes, the solution pH was adjusted to 7.0 using 0.1 M NaOH and 0.1 M HCl. CR and sulfamethoxazole (SMX) were sequentially added to the bottles, followed by the introduction of an oxidant solution to initiate the reaction. The reactors were then placed in a thermostatic shaker (YQ-600, Shanghai Hetian Scientific Instrument Co., Shanghai, China) at 200 rpm and 25 °C, and a liquid sample was withdrawn at each preselected time interval. Each sample was filtered through a 0.22 μm filter to determine the residual concentrations of pollutants and oxidants. To terminate the reaction, 10 μL of ascorbic acid or sodium thiosulfate was immediately added. To eliminate the influence of adsorption, desorption experiments were performed using a mixture of methanol and 1 M NaHCO_3_ in a 10:1 (*v*/*v*) ratio, achieving 95% recovery of SMX from the CR surface [2].

### 3.5. Analytical Methods

The functional groups were analyzed by Fourier transform infrared spectroscopy (FTI-IR; HORIBA EMAX, Japan), which was used to analyze the changes in the surface functional groups of the CR. The surface area, pore size and pore volume were determined by a specific surface analyzer (ASAP2020M, USA) at 77 K. The Raman spectra of CR were obtained using a Raman system at an excitation wavelength of 532 nm. The contents of C, H, O, N, and S in the CR were determined using a Vario EL II Elemental Analyzer (Elementar, Hanau, Germany). Oxygen content was determined using a mass balance according to previous studies. The contact angle of CR was determined by a contact angle measuring instrument (OCA20, Dataphysics, Filderstadt, Stuttgart, Germany). The generated free radical species were monitored by electron spin resonance (Bruker EMXPLUS10/12, Germany) at room temperature, with a resonance frequency of 9.84 GHz, microwave power of 20.00 mW, modulation frequency of 100.00 kHz, and modulation amplitude of 2.00 G. To measure the persistent free radicals (PFRs), 50 mg of CR was enclosed in a quartz capillary and analyzed by EPR (Bruker EMXplus, Germany). The XPS spectra were processed using CasaXPS 5.0 software, with background subtraction carried out prior to peak fitting. The full width at half maximum (FWHM) of all fitted components was constrained to ≤2 eV.

The concentration of PDS was determined using a UV-vis spectrophotometer (UV-1900, Shimadzu, Kyoto, Japan) in combination with potassium iodide. The concentration of SMX was monitored using a high-performance liquid chromatograph (HPLC-2030C, Shimadzu, Kyoto, Japan) equipped with a diode array detector and a Zorbax SB-C18 column (4.6 × 150 mm, 5 μm particle size) maintained at 30 °C. The mobile phase consisted of methanol and phosphoric acid (0.1%, *w*/*w*), and a gradient elution program was applied as follows: the methanol proportion was held at 10% for the first 1 min, then increased to 50% from 1 to 6 min, and finally decreased back to 10% from 10 to 15 min. The peak area of SMX was measured at a detection wavelength of 254 nm.

## 4. Conclusions

The introduction of iron sludge led to significant alterations in the characteristics of the resulting carbon residue (CR). This study demonstrates that co-pyrolysis of iron sludge and biochar can produce a promising material for adsorption and catalysis. Compared with other CRs, I/B 0.7 exhibited notable efficacy in pollutant adsorption, oxidant activation, and pollutant degradation, positioning it as an excellent functional material. The degradation of pollutants was primarily mediated by singlet oxygen (^1^O_2_) and hydroxyl radicals (·OH). The generation of reactive oxygen species was attributed to the presence of functional groups, persistent free radicals, and iron species. Furthermore, the I/B 0.7/PDS system demonstrated great potential for wastewater treatment, achieving highly efficient performance. The application of CR did not introduce any toxic substances into the aqueous matrix. Thus, this work not only presents the development of an effective material but also highlights the promising utilization of iron sludge as an environmentally friendly resource for sustainable wastewater treatment.

## Data Availability

Data will be made available on request.

## Supplementary Materials

The following supporting information can be downloaded at: https://www.mdpi.com/article/10.3390/molecules31132374/s1. Table S1: The reaction rate of MB adsorption. Table S2: The reaction rate of the biochar/PDS system. Table S3: The specific surface area and pore size of I/B 0.7. Table S4: The elemental content of I/B 0.7. Table S5: The physical and chemical properties of biochar. Table S6: The change in persistent free radical content of biochar. Figure S1: The adsorption of SMX by CRs. Figure S2: The FTIR results of I/B 0.7. Figure S3: The SMX degradation (a) and PDS activation (b) in the I/B 0.7/PDS system after the quenching of functional groups. Figure S4: The SMX degradation (a) and PDS activation (b) in the I/B 0.7/PDS system treated with KI. Figure S5: The Roman results of I/B 0.7. Figure S6: The SMX degradation (a) and PDS activation (b) in the I/B 0.7/PDS system treated with EDTA-2Na. Figure S7: The leaching content of Fe in the I/B 0.7/PDs system. Figure S8: The bacterial luminescence result in the I/B 0.7 matrix.
