# Supplementary material for: Dual-Functional Carbon Residue Derived from Co-Pyrolysis of Iron Sludge and Biochar for Synergistic Adsorption and Catalytic Oxidation
Source: Molecules. 2026 Jul 6;31(13):2374. doi: 10.3390/molecules31132374 (PMC13362782; doi:10.3390/molecules31132374)
Supplement: Supplementary file 1 [file molecules-31-02374-s001.zip › molecules-4354961-supplementary.pdf]

Supporting Information

Dual-functional Carbon Residue Derived from Co-pyrolysis of Iron Sludge and Biochar for Synergistic Adsorption and Catalytic Oxidation

Zhipeng Li[2,3]; Gangzheng Sun[2]; Hao Zhang[1*]; Yiwei Xiang[2]; Weikun Zhang[2]; Guoying Pang[1]; Siyu Wei[1]; Nanxiang Deng[1]; Tan Meng[3*]

1. School of Mechanical and Power Engineering, Tianjin Renai College, Tianjin 301636, China

2. Shengli Oilfield Company, Sinopec, Dongying, 257000, China;

3. School of Environmental Science and Engineering, Tianjin University, Tianjin 300072, China;

Corresponding authors: *Hao Zhang; Email: 15130248376@163.com

*Tan Meng; Email: tanmeng@tju.edu.cn

Number of pages: 15

8 Figures, 6 Tables

Table S1. The reaction rate of MB adsorption.

	MB adsorption (min^{-1})		MB adsorption (min^{-1})
I/S 0	0.0798	I/B 0	0.1396
I/S 0.2	0.0633	I/B 0.2	0.1658
I/S 0.7	0.04	I/B 0.7	0.1696
I/S 1	0.0428	I/B 1	0.1636
I/S 2	0.0321	I/B 2	0.1621
Iron sludge	0.0154		

Table S2. The reaction rate of biochar/PDS system.

	SMX adsorption (min^{-1})	SMX degradation (min^{-1})	PDS decomposition (min^{-1})
I/S 0	0.0216	0.0062	0.0035
I/S 0.7	0.0136	0.0059	0.0018
I/B 0.7	0.0246	0.0184	0.0075
Iron sludge	0.0003	0.0012	0.0003

Table S3 The specific surface area and pore size of I/B 0.7.

	**Specific surface area (m²/g)**	**Pore volume cm³/g)**	**Pore size (nm)**
**I/B 0.7**	441.2	0.241	2.188
**I/B 0.7 after adsorption**	382.5	0.205	1.825

Table S4 The elemental content of I/B 0.7.

	C	N	O	Fe	S	Si
**I/B 0.7**	79 %	1 %	9 %	1 %	6 %	3 %
**I/B 0.7 after adsorption**	81 %	1 %	5 %	1 %	9 %	3 %

Table S5. The physical and chemical properties of biochar.

	**Contact angle（°）**	**pH_{pzc}**
**I/S 0**	126.07	9.46
**I/S 0.7**	125.95	8.89
**I/B 0.7**	94.43	9.87

Table S6. The change of persistent free radicals content of biochar.

	g factor	PFRs concentration (10^5 spins/g)
Control	2.0045	6.052
KI treatment		4.019

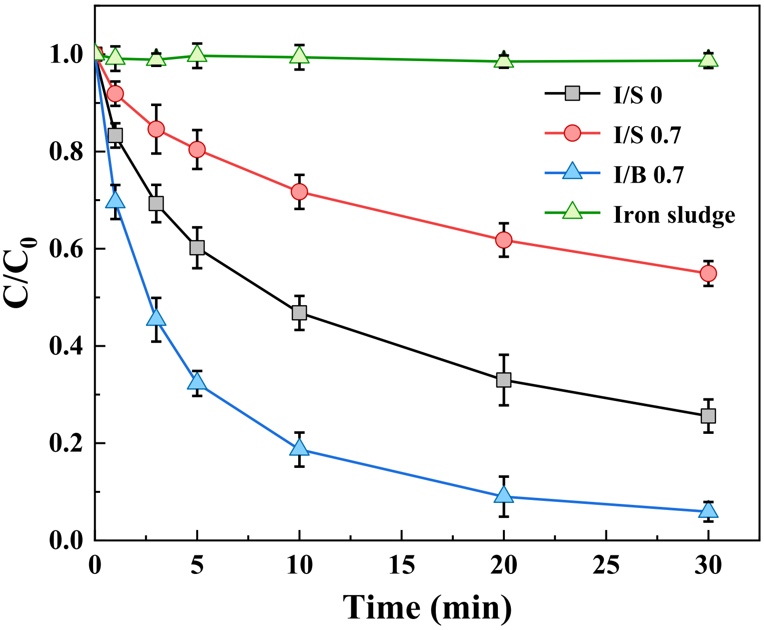

Figure S1. The adsorption of SMX by CRs. [CR] = 1 g/L, [SMX] = 10 uM, pH = 7.

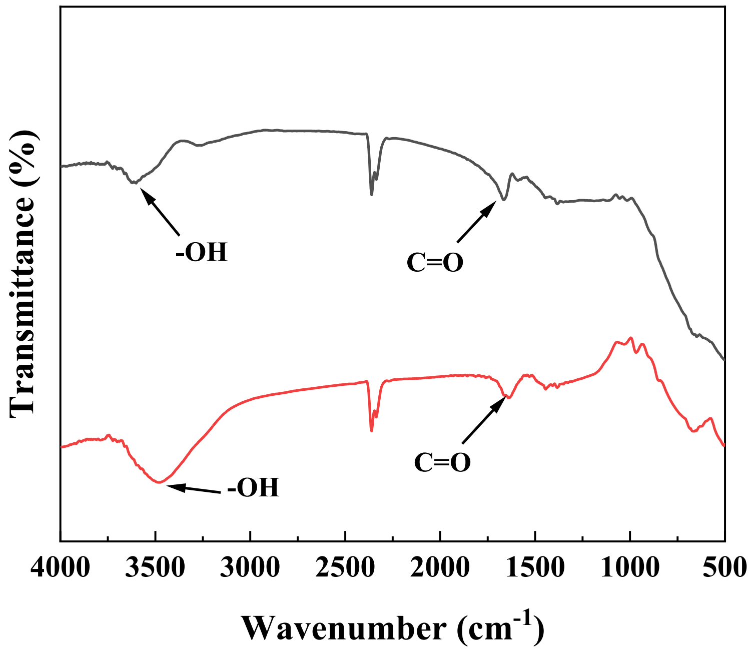

Figure S2. The FTIR results of I/B 0.7 (Red line: Before reaction; Black line: After reaction).

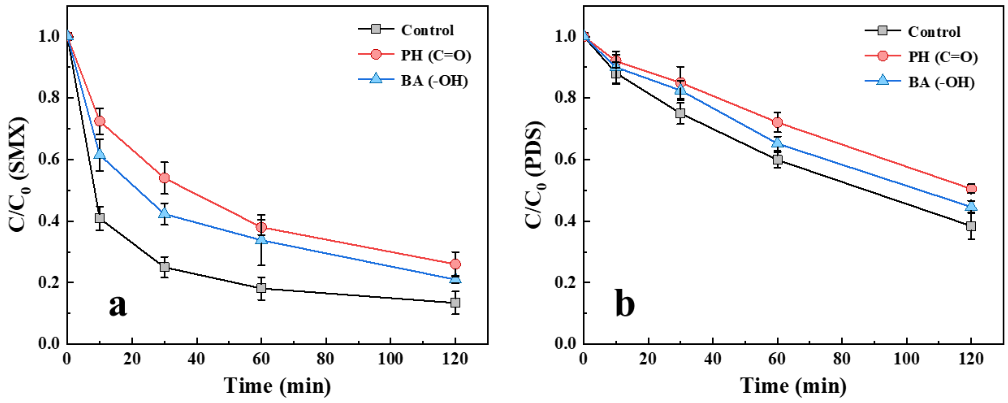

Figure S3. The SMX degradation (a) and PDS activation (b) in I/B 0.7/PDS system after the quenching of functional groups. [I/B 0.7] = 1g/L, [PDS] = 10 mM, [SMX] = 10 μM, pH = 7.

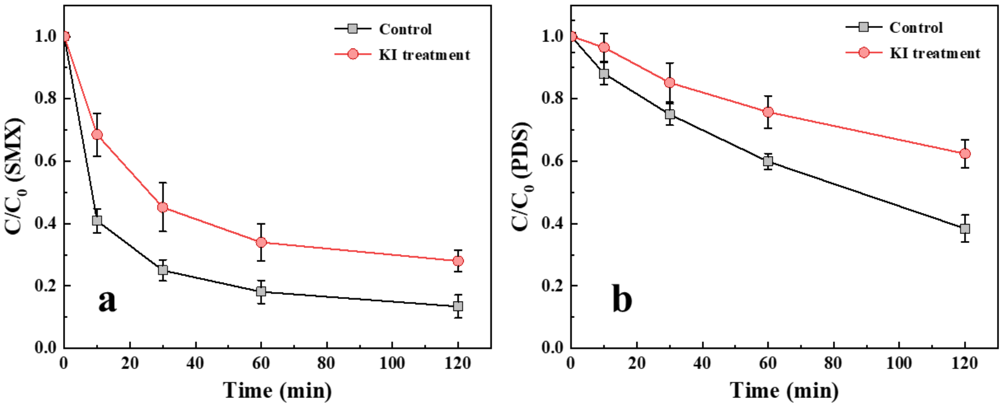

Figure S4. The SMX degradation (a) and PDS activation (b) in I/B 0.7/PDS system treated by KI. [I/B 0.7] = 1g/L, [PDS] = 10 mM, [SMX] = 10 μM, pH = 7.

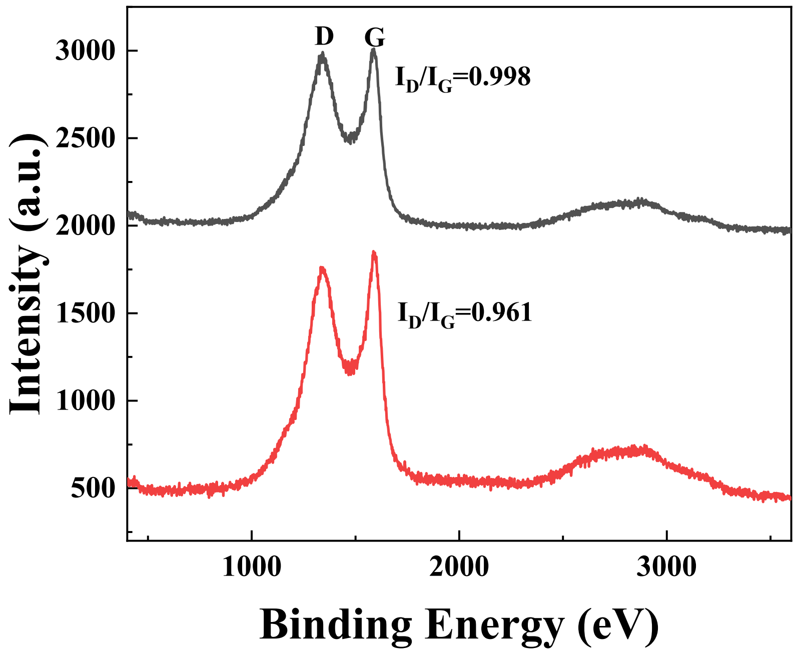

Figure S5. The Roman results of I/B 0.7 (Black line: before reaction; Red line: after reaction).

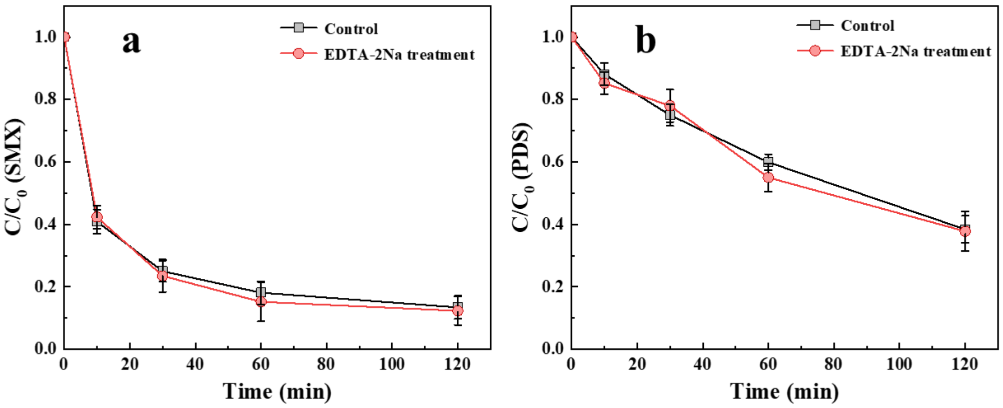

Figure S6. The SMX degradation (a) and PDS activation (b) in I/B 0.7/PDS system treated by EDTA-2Na. [I/B 0.7] = 1g/L, [PDS] = 10 mM, [SMX] = 10 μM, pH = 7.

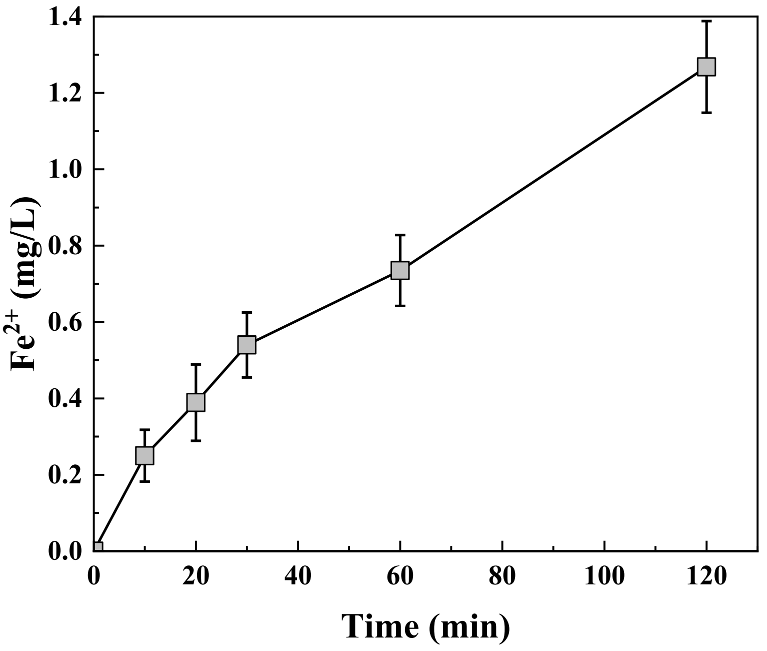

Figure S7. The leaching content of Fe in I/B 0.7/PDs system. [I/B 0.7] = 1g/L, [PDS] = 10 mM, [SMX] = 10 μM, pH = 7.

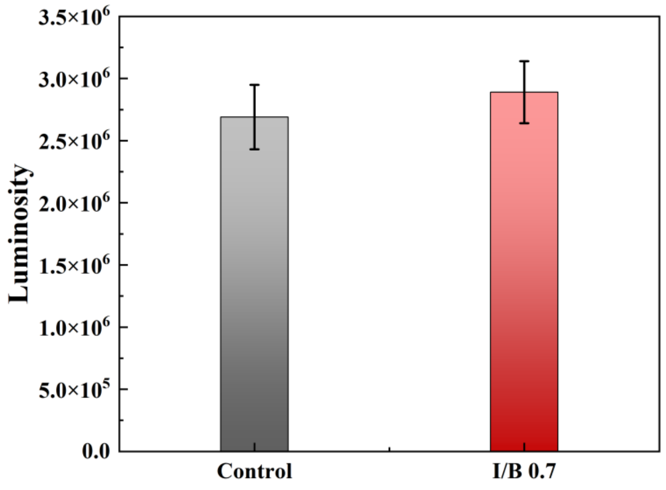

Figure S8. The bacterial luminescence result in I/B 0.7 matrix. [I/B 0.7] = 1g/L, [PDS] = 10 mM, [SMX] = 10 μM, pH = 7.
